# Dysfunctional ABCG2 gene polymorphisms are associated with serum uric acid levels and all-cause mortality in hemodialysis patients

**DOI:** 10.1007/s13577-020-00342-w

**Published:** 2020-03-16

**Authors:** Akio Nakashima, Kimiyoshi Ichida, Ichiro Ohkido, Keitaro Yokoyama, Hirotaka Matsuo, Yuki Ohashi, Tappei Takada, Akiyoshi Nakayama, Hiroshi Suzuki, Nariyoshi Shinomiya, Mitsuyoshi Urashima, Takashi Yokoo

**Affiliations:** 1 Division of Nephrology and Hypertension, Department of Internal Medicine, Jikei University School of Medicine, Tokyo, Japan; 2 Department of Pathophysiology, Tokyo University of Pharmacy and Life Sciences, 1432-1 Horinouchi, Hachioji, Tokyo 192-0392 Japan; 3 Department of Integrative Physiology and Bio-Nano Medicine, National Defense Medical College, Tokorozawa, Japan; 4 Department of Pharmacy, The University of Tokyo Hospital, Faculty of Medicine, The University of Tokyo, Tokyo, Japan; 5 Division of Molecular Epidemiology, Jikei University School of Medicine, Tokyo, Japan

**Keywords:** Uric acid, Hemodialysis, ABCG2, Polymorphism, Mortality

## Abstract

Dysfunctional variants of ATP-binding cassette transporter subfamily G member 2 (ABCG2), a urate transporter in the kidney and intestine, are the major causes of hyperuricemia and gout. A recent study found that ABCG2 is a major transporter of uremic toxins; however, few studies have investigated the relationship between ABCG2 gene polymorphisms and mortality. This prospective cohort study of 1214 hemodialysis patients investigated the association between serum uric acid levels and ABCG2 genotype and mortality. Genotyping of dysfunctional ABCG2 variants, Q126X (rs72552713) and Q141K (rs2231142), was performed using the patients’ DNA. During the study period, 220 patients died. Lower serum uric acid levels were associated with higher mortality (hazard ratio [HR] 1.89, 95% confidence interval [CI] 1.14–3.10, *P* ≤ 0.001). ABCG2 dysfunction, estimated by genetic variants, had a significant positive association with serum uric acid levels (full function: 7.4 ± 1.2 mg/dl, 3/4 function: 7.9 ± 1.3 mg/dl, 1/2 function: 8.2 ± 1.4 mg/dl, ≤ 1/4 function: 8.7 ± 1.3 mg/dl, *P* ≤ 0.001). This association remained significant on multiple regression analysis. The Cox proportional hazard analysis indicated that the ABCG2 ≤ 1/4 function type was significantly associated with higher mortality (HR 6.66, 95% CI 2.49 to 17.8, *P* ≤ 0.001) than the other function types. These results showed that ABCG2 plays a physiologically important role in uric acid excretion, and that ABCG2 dysfunction is a risk factor for mortality in hemodialysis patients.

**Electronic supplementary material:**

The online version of this article (10.1007/s13577-020-00342-w) contains supplementary material, which is available to authorized users.

## Introduction

ATP-binding cassette transporter subfamily G member 2 (ABCG2) is located in a gout-susceptibility locus on chromosome 4q, which was identified by previous studies including genome-wide association studies of serum uric acid levels [1, 2]. ABCG2 is a high-capacity urate exporter in the kidney and intestine, the dysfunction of which increases the risk for gout and hyperuricemia [3–6]. Since uric acid, the final product of purine metabolism, is mainly excreted by the kidneys, hyperuricemia is a common condition in patients with kidney failure [7, 8]. In addition, hyperuricemia is a risk factor for cardiovascular disease (CVD) in the general population [9, 10]. Furthermore, a previous study reported that serum uric acid levels were dependent on ABCG2 polymorphisms [3, 6, 11]. These results suggest that ABCG2 plays a prominent role in uric acid homeostasis.

We recently found that ABCG2 is a major transporter of the uremic toxin indoxyl sulfate [12]. ABCG2-knockout mice had lower survival rates and higher plasma indoxyl sulfate levels during kidney dysfunction [12]. In addition, several studies have also reported that ABCG2 plays an important role in stimulating inflammation and regulating autophagy [13−15]. ABCG2 dysfunction may affect clinical outcomes, such as all-cause mortality, through accumulation of uric acid and uremic toxins and acceleration of inflammation and suppression of autophagy. However, no studies have analyzed the associations between ABCG2 polymorphism and clinical outcomes. Dialysis patients are usually anuric, thus the effect of renal excretion on uremic toxins is relatively small. For the above reasons, it is necessary to evaluate the influence of ABCG2 dysfunction in the intestine on clinical outcomes of dialysis patients. The aim of the present study was to investigate the association between serum uric acid levels and mortality in hemodialysis patients and to clarify the effects of estimated ABCG2 function on serum uric acid levels and mortality.

## Methods

### Study design

In this prospective multicenter cohort study, hemodialysis patients were recruited at the dialysis outpatient units of 15 medical institutions in Tokyo, Japan [16, 17]. Baseline visits for patient enrollment were conducted between May 1, 2011 and March 31, 2012, and enrolled patients were followed up to June 1, 2016. Patients were older than 20 years, had spent at least 3 months on dialysis therapy, and regularly received thrice-weekly hemodialysis (3–5 h/session). Patients with acute gastrointestinal bleeding, acute coronary syndrome, and liver dysfunction at baseline were excluded. The study protocol was reviewed and approved by the ethics committee of the Jikei Institutional Review Board at Jikei University School of Medicine (22-182 6359). This study was also approved by each participating institution’s review board. All study procedures were in accordance with the Declaration of Helsinki and its revisions. Written informed consent was obtained from all patients prior to inclusion in the study.

### Data collection

Age, body mass index (BMI), sex, dialysis vintage, primary illness leading to kidney dysfunction, and past medical history were extracted from medical records. Medication information (use of anti-hyperuricemic drugs including allopurinol and febuxostat, antiplatelet drugs, vitamin K antagonists, phosphate binders, vitamin D receptor agonists, cinacalcet, antihypertensive medications, and statins) was obtained from prescription records. Comorbidities and medications were determined by chart review and standardized interviews at baseline.

Blood samples were collected at study entry, before the hemodialysis session conducted after the longest inter-dialysis period. Routine biochemical measurements included serum creatinine, uric acid, sodium, potassium, phosphorus, calcium, serum albumin, blood urea nitrogen (BUN), alkaline phosphatase, hematocrit, intact parathyroid hormone (PTH), and C-reactive protein (CRP) levels. The delivered dialysis dose was measured by single pool Kt/V.

### Outcomes

Clinical outcomes were prospectively recorded and coded, and blinded from clinical and biochemical data. This information was collected by study investigators. After review of the available information, the cause of death was classified as CVD, infectious disease, malignancy, or other. The primary outcome was all-cause mortality. In all analyses, follow-up was censored at loss to follow-up, renal transplantation, or the end of the study.

### Statistical analysis

Non-normally distributed data are presented as medians (25th and 75th percentiles), and normally distributed data are summarized as means ± standard deviation (SD). Binary data are summarized as percentages. Differences between more than two groups were analyzed by analysis of variance or the Kruskal–Wallis test, as appropriate. Nominal variables were analyzed by the *χ*^2^ test. Patient characteristics were described by uric acid levels (quartiles). Serum uric acid levels were categorized by ABCG2 functions and use of anti-hyperuricemic drugs. To clarify the serum uric acid levels by ABCG2 functions, multiple regression analysis was used. Age, dialysis vintage, BMI, systolic blood pressure, diabetes mellitus, hemoglobin, albumin, BUN, creatinine, potassium, calcium, phosphorus, CRP, Kt/V, angiotensin covering enzyme inhibitors, angiotensin II receptor blockers, statins, antiplatelet drugs, and anti-hyperuricemic drugs were adopted as covariates.

Cox proportional hazard models were used to investigate the association between all-cause mortality and uric acid levels. Age, dialysis vintage, sex, diabetes mellitus, BMI, systolic blood pressure, hemoglobin, albumin, creatinine, potassium, calcium, phosphate, CRP, Kt/V, past history of CVD events, use of angiotensin-converting enzyme inhibitors or angiotensin II receptor blockers, use of statins, use of vitamin D receptor agonists, use of antiplatelet drugs, and use of anti-hyperuricemic drugs were adopted as covariates. An adjusted restricted cubic spline curve with three knots was generated to show the non-linear association with all-cause mortality, and serum uric acid as a continuous variable was also examined using a fully adjusted model.

To investigate the association between ABCG2 function and all-cause mortality, Kaplan–Meier survival curves and log-rank tests were used, as well as a Cox proportional hazard model. Univariate and multivariate analyses are presented as [hazard ratio (HR); 95% confidence interval (CI)]. The following covariates were used for Cox proportional hazard models. Model I included age, dialysis vintage, sex, diabetes mellitus, BMI, systolic blood pressure, hemoglobin, albumin, creatinine, potassium, calcium, phosphate, CRP, Kt/V, past history of CVD, use of angiotensin-converting enzyme inhibitors or angiotensin II receptor blockers, use of statins, use of vitamin D receptor agonists, and use of antiplatelet drugs. Model II included all covariates of Model I, uric acid, and use of anti-hyperuricemic drugs. Significance was set at *P* < 0.05. All statistical analyses were performed using STATA 13.0 (STATA Corp., College Station, TX, USA).

### Genetic analysis and assessment of ABCG2 function

Genomic DNA was extracted from whole peripheral blood cells. Genotyping of ABCG2 dysfunctional variants (Q126X and Q141K) was performed using the TaqMan method (Life Technologies Corporation, Carlsbad, CA, USA) with a Light Cycler 480 (Roche Diagnostics, Mannheim, Germany), as previously described [11, 18]. Custom TaqMan assay probes were designed as follows: for Q126X, VIC-CCACTAATACTTACTTGTACCAC and FAM-CCACTAATACTTACTTATACCAC; and for Q141K, VIC-CTGCTGAGAACTGTAAGTT and FAM-CTGCTGAGAACTTTAAGTT.

To confirm their genotypes, DNA sequencing analysis was performed with the following primers: for Q126X, forward 5′-TGTACAATGAAAAGAGAAAGGTGAG-3′ and reverse 5′-CTGCCTTTTCACATAAGTGTC-3′; and for Q141K, forward 5′-ATGGAGTTAACTGTCATTTGC-3′ and reverse 5′-CACGTTCATATTATGTAACAAGCC-3′. PCR products were sequenced with the ABI PRISM 3700 Genetic Analyzer (Applied Biosystems, Foster City, CA, USA). We previously reported that Q126X is a nonfunctional variant, Q141K is a half-functional variant for urate excretion compared to the wild-type, and there was no simultaneous presence of the minor alleles of Q126X and Q141K in one haplotype [3, 5]. Thus, three haplotypes were defined as *1 (126Q and 141Q), *2 (126Q and 141 K), and *3 (126X and 141Q), and all patients could be divided into the following ABCG2 functional groups: full function (*1/*1), 3/4 function (mild dysfunction, *1/*2), 1/2 function (moderate dysfunction, *1/*3 or *2/*2), and ≤ 1/4 function (severe dysfunction, *2/*3 or *3/*3) [18].

## Results

### Patient characteristics categorized by serum uric acid levels

A total of 1214 hemodialysis patients were evaluated and divided into quartiles according to serum uric acid levels (Table 1). Significantly higher age and lower levels of BMI, serum BUN, serum creatinine, and serum phosphate were observed in patients with low serum uric acid levels. The prevalence of anti-hyperuricemic drug use was significantly higher in the low uric acid level group. In addition, a past history of CVD was more common in patients with low serum uric acid levels. On the other hand, there were no significant differences in dialysis vintage, CRP, and use of antiplatelet drugs among the four groups.

**Table 1:** Patient characteristics

Serum uric acid level (mg/dl)	Quartile 1	Quartile 2	Quartile 3	Quartile 4	*P* value
	UA < 6.8	6.8 ≤ UA < 7.6	7.6 ≤ UA < 8.5	UA ≥ 8.5	
Number	297	332	273	312	
Age (years)	67 (11.5)	64 (11.2)	63 (10.9)	60 (12.2)	< 0.001
Male (%)	212 (71.4)	236 (71.1)	182 (66.7)	218 (69.9)	0.6
Dialysis vintage (months)	80 (34–151)	88 (40–165)	85 (42–140)	93 (43–149)	0.14
Diabetes mellitus (%)	139 (46.8)	114 (34.3)	106 (38.8)	101 (32.4)	< 0.001
Body mass index (kg/m^2^)	21.2 (4.5)	21.0 (5.2)	22.2 (5.1)	22.6 (5.1)	< 0.001
sBP (mmHg)	153 (23)	152 (22)	151 (21)	152 (21)	0.64
dBP (mmHg)	78 (14)	80 (13)	80 (15)	80 (14)	0.07
Hemoglobin (g/dl)	10.3 (1.0)	10.5 (1.0)	10.5 (1.0)	10.6 (1.1)	0.02
Albumin (g/dl)	3.69 (0.34)	3.76 (0.36)	3.77 (0.33)	3.81 (0.36)	< 0.001
Blood urea nitrogen (mg/dl)	58 (13)	63 (14)	67 (13)	72 (13)	< 0.001
Creatinine (mg/dl)	10.4 (2.6)	11.4 (2.9)	11.8 (3.0)	12.7 (3.4)	< 0.001
Sodium (mEq/l)	139 (3)	139 (3)	139 (3)	139 (3)	0.32
Potassium (mEq/l)	4.92 (0.71)	5.02 (0.74)	5.03 (0.69)	5.02 (0.67)	< 0.001
ALP (IU/l)	225 (176–284)	213 (171–271)	221 (176–282)	212 (168–276)	0.03
Calcium (mg/dl)	8.9 (0.6)	8.9 (0.7)	8.9 (0.6)	8.9 (0.6)	0.95
Phosphate (mg/dl)	5.0 (1.2)	5.4 (1.4)	5.6 (1.4)	5.8 (1.4)	< 0.001
Magnesium (mg/dl)	2.6 (0.5)	2.6 (0.4)	2.6 (0.4)	2.6 (0.5)	0.57
iPTH (pg/ml)	134 (82–209)	140 (79–232)	148 (80–205)	168 (100–261)	< 0.001
C-reactive protein (mg/dl)	0.1 (0.05–0.32)	0.1 (0.05–0.3)	0.1 (0.05–0.5)	0.1 (0.06–0.38)	0.27
Kt/V	1.4 (0.7)	1.4 (0.3)	1.4 (0.3)	1.4 (0.3)	0.52
Anti-hyperuricemic drug (%)	64 (21.6)	56 (16.9)	46 (16.9)	32 (10.3)	< 0.001
Antiplatelet drug (%)	152 (51.2)	155 (16.7)	137 (50.2)	135 (43.3)	0.2
Anticoagulant drug (%)	24 (8.1)	32 (9.6)	15 (5.5)	21 (6.7)	0.25
ACE-I or ARB (%)	155 (53.3)	160 (49.5)	122 (45.7)	161 (53.1)	0.23
Statin (%)	92 (31)	80 (24.1)	74 (27.1)	67 (21.5)	0.05
Past history					
Cardiovascular disease (%)	66 (22.2)	52 (15.7)	61 (22.3)	43 (13.8)	0.01
Malignancy (%)	70 (23.6)	56 (16.9)	38 (13.9)	38 (12.2)	< 0.001

Data are means (SD), N (%), or medians (interquartile range) as appropriate

*UA* uric acid, *sBP* systolic blood pressure, *dBP* diastolic blood pressure, *ALP* alkaline phosphatase, *iPTH* intact parathyroid hormone, *ACE-I* angiotensin-converting enzyme inhibitor, *ARB* angiotensin II receptor blocker

### Serum uric acid levels and mortality

**Fig. 1:**
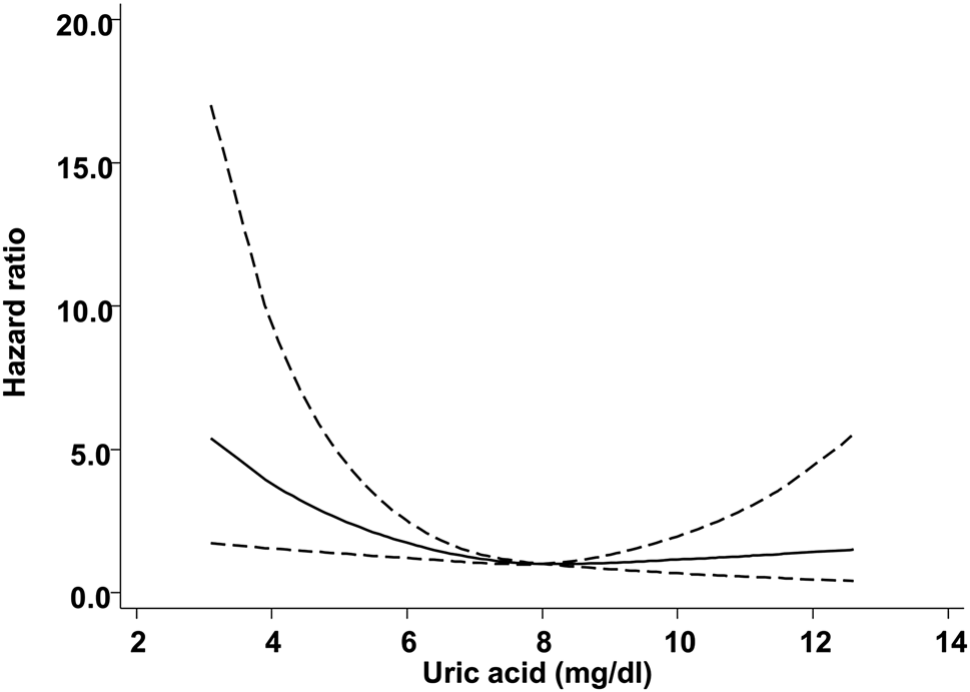
Restricted cubic spline curve of serum uric acid levels for all-cause mortality. The spline model has three degrees of freedom. The multivariate model includes age, dialysis vintage, sex, diabetes mellitus, body mass index, systolic blood pressure, hemoglobin, albumin, creatinine, potassium, calcium, phosphate, C-reactive protein, *Kt*/*V*, past history of cardiovascular disease events, use of angiotensin-converting enzyme inhibitors or angiotensin II receptor blockers, use of statins, use of vitamin D receptor agonists, use of antiplatelet drugs, and use of anti-hyperuricemic drugs. The solid line shows the hazard ratio for mortality. Dotted lines show the 95% confidence interval of the hazard ratio. Lower levels of serum uric acid are associated with higher mortality

During the follow-up period, 220 deaths occurred. Details of the events were as follows: CVD events 114; infectious diseases 52; malignancies 33; and others 21. Cox proportional hazard models were used to analyze the association between mortality and serum uric acid levels. All-cause mortality was significantly higher in the group with the lowest serum uric acid level, relative to the other groups in unadjusted models (HR 1.94, 95% CI 1.33–2.81, *P* ≤ 0.001) and fully adjusted models (HR 1.89, 95% CI 1.14–3.10, *P* ≤ 0.001) (Table 2). There were no significant differences among other groups. The restricted cubic spline curve of serum uric acid levels also showed an increased risk of mortality with decreased serum uric acid levels (Fig. 1).

**Table 2:** Cox hazard regression analysis for all-cause mortality by serum uric acid level

Serum uric acid level (mg/dl)	Quartile 1	Quartile 2	Quartile 3	Quartile 4	
	UA < 6.8	6.8 ≤ UA < 7.6	7.6 ≤ UA < 8.5	UA ≥ 8.5	
Number	297	332	273	312	
Number of events (*n*)	80	55	42	43	
Unadjusted HR (95% CI)	1.94 (1.33–2.81)	1.08 (0.72–1.61)	1.0 (Reference)	0.89	(0.58–1.36)
*P *value	< 0.001	0.71		0.61	
Adjusted HR (95% CI)	1.89 (1.14–3.10)	1.21 (0.70–2.06)	1.0 (Reference)	1.21	(0.70–2.10)
*P *value	< 0.001	0.7		0.15	

Adjusted for age, dialysis vintage, sex, diabetes mellitus, body mass index, systolic blood pressure, hemoglobin, albumin, creatinine, potassium, calcium, phosphate, c-reactive protein, Kt/V, past history of cardiovascular disease event, use of angiotensin-converting enzyme inhibitors or angiotensin II receptor blockers, use of statins, use of vitamin D receptor agonists, use of anti-platelet drugs, and use of anti-hyperuricemic drugs

*UA* uric acid, *HR* hazard ratio, *CI* confidence interval

### ABCG2 function types and serum uric acid levels

**Fig. 2:**
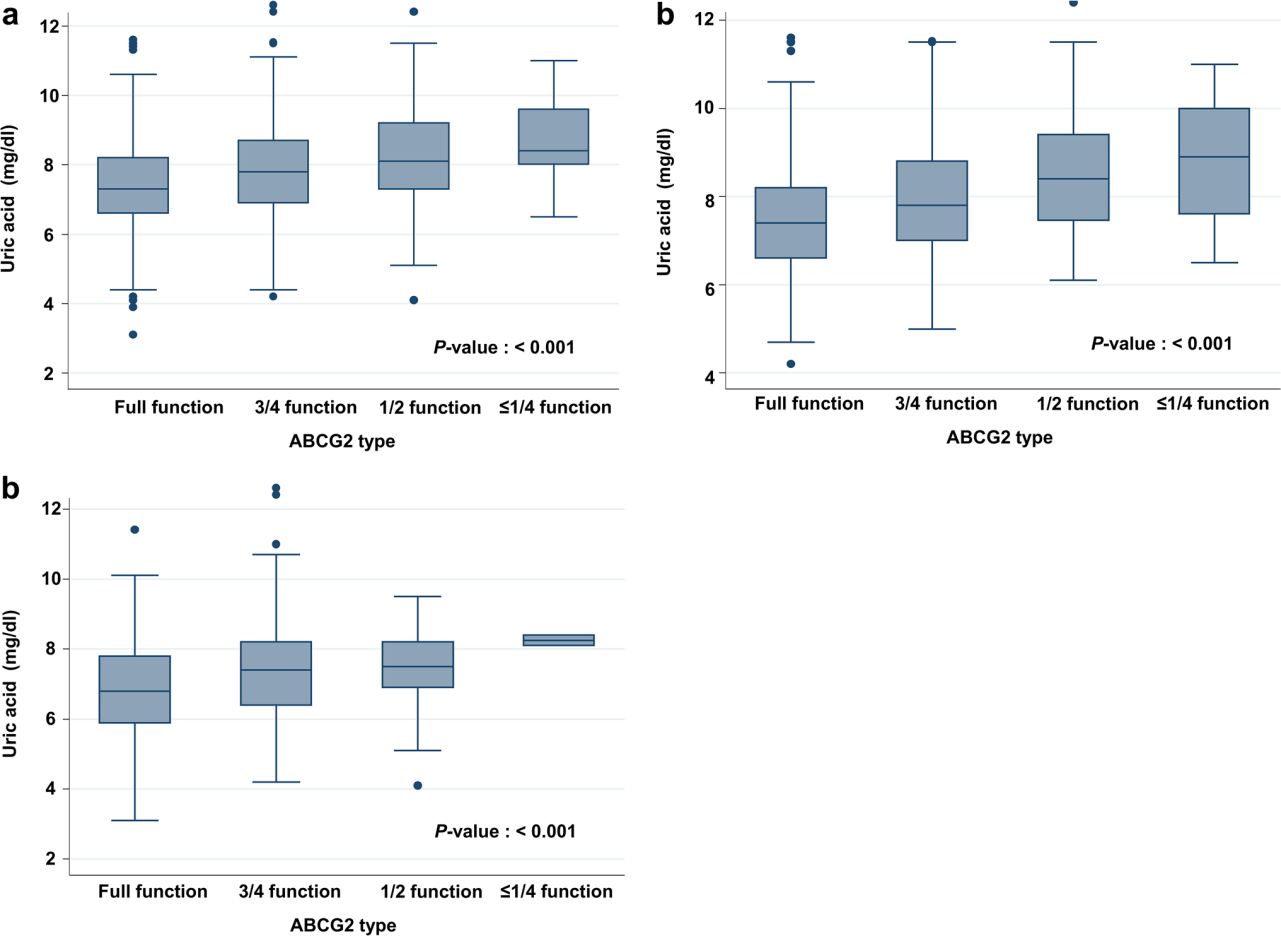
Serum uric acid levels by ABCG2 function and anti-hyperuricemic drug use. Associations between estimated ABCG2 function and serum uric acid levels are shown using all patients (**a**), non-users of anti-hyperuricemic drug (**b**), and anti-hyperuricemic drug users (**c**). The central box extends from the 25th to the 75th percentile. A center line within the box marks represents the median. Whiskers above and below the box indicate the 10th and 90th percentiles. All dots outside this range are outliers, which are not typical of the rest of the data. Statistical analysis was performed by analysis of variance

The number of patients categorized by estimated ABCG2 function types was as follows: full function 568 (46.8%); 3/4 function 504 (41.5%); 1/2 function 129 (10.7%); and ≤ 1/4 function 13 (1.0%) (Table 3). A set of important demographic and clinical data divided by ABCG2 function were included as seen in Supp 1. Serum uric acid levels were lowest in the full function group, and they gradually increased in accordance with the decrease in estimated ABCG2 function (mean ± SD, full function: 7.4 ± 1.2 mg/dl, 3/4 function: 7.9 ± 1.3 mg/dl, 1/2 function: 8.2 ± 1.4 mg/dl, ≤ 1/4 function: 8.7 ± 1.3 mg/dl, *P* ≤ 0.001) (Fig. 2a). Serum uric acid levels were also analyzed by estimated ABCG2 function categorized by anti-hyperuricemic drugs. In this study, 198 patients (16.3%) were prescribed anti-hyperuricemic drugs. As shown in Fig. 2b, c, the effect of estimated ABCG2 function was not modified by the use of anti-hyperuricemic drugs.

**Table 3:** Prevalence of estimated ABCG2 function types and genotype combinations

	Genotype combination		Number (%)
	Q126X(rs72552713)	Q141K(rs2231142)	
≤ 1/4 function	T/T	C/C	2 (0.1)
	T/C	C/A	11 (0.9)
1/2 function	T/C	C/C	36 (3.0)
	C/C	A/A	93 (7.7)
3/4 function	C/C	C/A	504 (41.5)
Full function	C/C	C/C	568 (46.8)
Total			1214

Using multiple linear regression analysis of uric acid levels with the dependent variables, estimated ABCG2 function was significantly associated with serum uric acid levels in the adjusted models (beta-coefficient ± SD, 3/4 function: 0.67 ± 0.08 *P* ≤ 0.001, 1/2 function: 1.05 ± 0.13 *P* ≤ 0.001, ≤ 1/4 function: 1.83 ± 0.36 *P* ≤ 0.001) (Table 4).

**Table 4:** Association between serum uric acid levels and ABCG2 function types as determined by univariate and multivariate regression analyses

ABCG2 function	Uric acid (mg/dl)	Univariate analysis		Multivariate analysis	
		Beta coefficient	*P* value	Beta coefficient	*P* value
Full function	7.4 ± 1.2	Ref		Ref	
3/4 function	7.9 ± 1.3	0.47 ± 0.08	< 0.001	0.67 ± 0.08	< 0.001
1/2 function	8.2 ± 1.4	0.83 ± 0.13	< 0.001	1.05 ± 0.13	< 0.001
≤ 1/4 function	8.7 ± 1.3	1.29 ± 0.36	< 0.001	1.83 ± 0.36	< 0.001

Adjusted for age, dialysis vintage, body mass index, systolic blood pressure, diabetes mellitus, hemoglobin, albumin, blood urea nitrogen, creatinine, potassium, calcium, phosphorus, C-reactive protein, Kt/V, angiotensin-converting enzyme inhibitor, angiotensin II receptor blocker, statins, anti-platelet drugs, and, anti-hyperuricemic drugs

### All-cause mortality and ABCG2 function

**Fig. 3:**
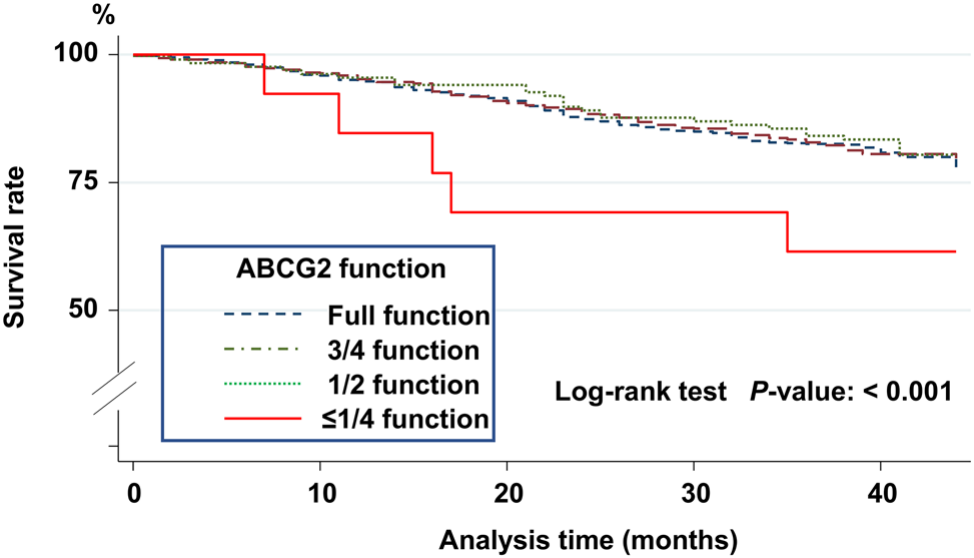
Kaplan–Meier survival analysis for all-cause mortality. During the follow-up period, 220 deaths occurred. The ABCG2 ≤ 1/4 function type was associated with significantly higher mortality than the other ABCG2 gene

During the study period, 220 patients died. The ABCG2 ≤ 1/4 function type showed a significantly higher mortality rate than the other function types with the log-rank test (*P* ≤ 0.001) (Fig. 3). The causes of death in patients with the ABCG2 ≤ 1/4 function type were CVD events 1, infection 3, and others 1. A Cox-proportional hazard analysis for all-cause mortality was also performed. In reference to the ABCG2 full-function type, the ≤ 1/4 function type was significantly associated with all-cause mortality in the unadjusted model (HR 2.55, 95% CI 1.04 to 6.28, *P* = 0.04) and the fully adjusted model including serum uric acid levels and use of anti-hyperuricemic drugs (HR 6.66, 95% CI 2.49 to 17.8, *P* ≤ 0.001) (Table 5).

**Table 5:** Cox hazard regression analysis of ABCG2 function types with all-cause mortality in hemodialysis patients

	Unadjusted model			Model I			Model II		
	HR	95% CI	*P *value	HR	95% CI	*P *value	HR	95% CI	*P *value
ABCG2 function									
Full function	Ref			Ref			Ref		
3/4 function	1.00	0.78 to 1.33	0.978	0.88	0.61 to 1.28	0.504	0.97	0.67 to 1.42	0.890
1/2 function	0.83	0.52 to 1.35	0.470	0.58	0.29 to 1.16	0.125	0.68	0.34 to 1.37	0.284
≤ 1/4 function	2.55	1.04 to 6.28	0.040	5.22	1.97 to 13.7	< 0.001	6.66	2.49 to 17.8	< 0.001

Model I: Adjusted for age, dialysis vintage, sex, diabetes mellitus, body mass index, systolic blood pressure, hemoglobin, albumin, creatinine, potassium, calcium, phosphate, c-reactive protein, Kt/V, past history of cardiovascular disease event, use of angiotensin-converting enzyme inhibitors or angiotensin II receptor blockers, statins, vitamin D receptor agonists, and anti-platelet drugs

Model II: Model I + (uric acid + anti-hyperuricemic drugs)

*HR* hazard ratio, *CI* confidence interval

## Discussion

In this study, lower serum uric acid levels were found to be associated with all-cause mortality in patients on hemodialysis. Estimated ABCG2 function was also found to be associated with serum uric acid levels, and this association was not modified by the use of anti-hyperuricemic drugs. Furthermore, this is the first study to show that severe ABCG2 dysfunction (≤ 1/4 function type) was associated with higher mortality than the other function levels in hemodialysis patients. This result was independent of serum uric acid levels and the use of anti-hyperuricemic drugs.

It has been suggested that hyperuricemia is linked to hypertension, insulin resistance, and metabolic syndrome [19]. Some studies have reported that hyperuricemia is a risk factor for CVD in the general population [9, 10]. However, whether or not hyperuricemia is a risk factor for CVD events and mortality in dialysis patients is controversial [7, 8, 20–22]. Recent studies reported that serum uric acid levels are positively correlated with already known laboratory nutritional markers (albumin, creatinine), body composition parameters, handgrip strength, and the geriatric nutritional risk index in hemodialysis patients [23, 24]. Although serum albumin levels, creatinine, and BMI were used as nutritional status indicators in the multivariate analysis of the present study, serum uric acid was significantly associated with mortality in the fully adjusted models. The present results indicated that serum uric acid levels reflect nutritional status, which is not evaluated by ordinary makers such as albumin. Because a previous study reported that serum uric acid remained a mortality risk factor after adjustment for nutritional markers, the normal protein catabolic rate (nPCR) [24] or subjective global assessment (SGA) [25], serum uric acid in dialysis patients is not regarded as a simple nutritional marker. Another mechanism of uric acid’s protective effect is related to antioxidants in human biologic fluids [26]. Although uric acid is reported to evoke oxidative stress thorough peroxynitrite and lipoprotein [27, 28], uric acid provides an antioxidant effect to reduce oxo-heme oxidant levels and protect erythrocytes from peroxidation [29]. Because patients on dialysis have higher oxidative stress, uric acid may have an important antioxidant effect. However, there have been no interventional studies to investigate whether elevation of uric acid attenuates the mortality risk in dialysis patients. Future studies are needed to evaluate the association between uric acid and mortality.

Since ABCG2 is the main urate transporter in both the kidney and intestine [3, 5, 11, 18, 30, 31], previous studies reported that dysfunctional ABCG2 gene polymorphisms are associated with hyperuricemia by decreasing urate excretion [3, 32]. In addition, ABCG2 is also associated with the prevalence and onset of gout [3, 6]. About two-thirds of urate is excreted from the kidneys and about one-third from the intestines [33]. ABCG2 knockout mice showed decreased intestinal excretion of uric acid [5, 34]. ABCG2-mediated intestinal urate excretion was also evidenced in a human study [11]. The present results showed that serum uric acid levels differed according to the estimated levels of ABCG2 function in hemodialysis patients. These results indicated that because patients on hemodialysis are usually anuric, the differences in serum uric acid levels between ABCG2 functions might depend on intestinal excretion. A previous study also reported that serum uric acid levels in dialysis patients are affected by ABCG2 gene types [11].

The present study is the first to show that ABCG2 dysfunction is associated with all-cause mortality in hemodialysis patients. In the supp 1, there are no significant differences between ABCG2 function and contributory factors for mortality such as, age, diabetes mellitus, BMI, albumin, BUN, creatinine, potassium, phosphate, and iPTH. In addition, although we set explanatory variables in Cox hazard regression analysis for all-cause mortality to include serum uric acid levels and anti-hyprturicemic drugs, the effects of ABCG2 function types for mortality significantly remained. According to the above results, we consider the effects of ABCG2 function for mortality are independent of serum uric acid levels. There are several possible mechanisms for the association between ABCG2 polymorphism and mortality. First, the ABCG2 transporter regulates not only uric acid, but also uremic toxins in chronic kidney disease (CKD) [12]. Our recent study showed that ABCG2-knockout CKD mice had higher mortality and decreased indoxyl sulfate excretion in the kidney and intestine [12]. Indoxyl sulfate is a protein-bound uremic toxin resulting from bacterial metabolism of dietary tryptophan and is reported to be associated with mortality in CKD and dialysis patients [35−37]. Since ABCG2 gene type was significantly associated with higher serum uric acid levels in the present study, indoxyl sulfate may also be higher in patients with ABCG2 ≤ 1/4 function; potentially accounting for the elevated mortality. Second, because ABCG2 is associated with inflammation, ABCG2 gene type may affect CVD events through inflammation. Gout is known as a risk factor for CVD and all-cause mortality [38, 39]. However, since a previous study showed that gout is significantly associated with CVD events even after adjustment for serum uric acid levels [40], this finding suggested that gout and inflammation induce CVD events. ABCG2, which is reported to be associated with the onset of gout according to genetic analysis [1, 3, 6], has an important role in inflammation through inhibition of oxidative stress and NF-κB [13, 41]. This mechanism is mediated by the role of ABCG2 in exporting urate, which affects pro-oxidants in the intracellular setting and inhibits promotion of mitochondrial dysfunction [42]. Third, ABCG2 has been reported to play an important role in autophagy. ABCG2-overexpressing cell lines were more resistant to stressors such as nutrient deprivation and ionizing radiation. On the other hand, knockdown of ABCG2 reduced autophagic activity in resistant cells [15]. In addition, nuclear factor E2-related factor 2 (Nrf2), a transcription factor involved in oxidative stress responses, is reported to be associated with ABCG2 expression [43]. The activation of the Nrf2 pathway has a cardioprotective effect [44], which may be mediated by NrF2-induced ABCG2 expression and its consequent promotion of autophagy.

The present study is the first to show that ABCG2 gene type is associated with serum uric acid levels and mortality in dialysis patients. Few studies have reported the associations between common gene variants and clinical outcomes including death in dialysis patients. For example, JAK3, which is a single nucleotide polymorphism (SNP) of the Janus kinase–signal transducer and activator of transcription (Jak-Stat) signal transduction pathway, was reported to be associated with CVD events in dialysis [45]. To date, the Klotho gene [46] and the VEGF gene [47] have been reported to be associated with clinical outcomes in dialysis patients. The present study showed that the ABCG2 gene type with ≤ 1/4 function was associated with higher mortality and a hazard ratio higher than for other common gene variants. To predict mortality of dialysis patients, stratification of patients with genetic risk factors will be meaningful in the future.

This study has several limitations. First, since this was an observational study, no causal relationships between ABCG2 gene type and serum uric acid levels and all-cause mortality could be proven. Second, meal information was not investigated. Uric acid levels are influenced by daily meal consumption and nutritional status. Third, serum uric acid levels were measured only at baseline. Fourth, information about a past history of gout was lacking. Previous studies reported that gout is a risk factor for CVD events independent of serum uric acid levels. Fifth, serum indoxyl sulfate levels were not measured. Because a previous study reported that ABCG2 gene type is associated with sulfate levels [12], a future study is needed to investigate the association between ABCG2 gene type and sulfate levels in patients with CKD.

In conclusion, low levels of serum uric acid were associated with higher mortality in hemodialysis patients. In addition, ABCG2 gene types were associated with serum uric acid levels and all-cause mortality in hemodialysis patients. A future prospective, interventional study is required to clarify the effects of ABCG2 function on serum uric acid levels and clinical events and to elucidate the underlying mechanisms.

## Electronic supplementary material

Below is the link to the electronic supplementary material.

Supplementary file1 (DOCX 21 kb)

## References

[1] Dehghan A, Kottgen A, Yang Q (2008). Association of three genetic loci with uric acid concentration and risk of gout: a genome-wide association study. Lancet.

[2] Kolz M, Johnson T, Sanna S (2009). Meta-analysis of 28,141 individuals identifies common variants within five new loci that influence uric acid concentrations. PLoS Genet.

[3] Matsuo H, Takada T, Ichida K (2009). Common defects of ABCG2, a high-capacity urate exporter, cause gout: a function-based genetic analysis in a Japanese population. Sci Transl Med.

[4] Matsuo H, Ichida K, Takada T (2013). Common dysfunctional variants in ABCG2 are a major cause of early-onset gout. Sci Rep.

[5] Ichida K, Matsuo H, Takada T (2012). Decreased extra-renal urate excretion is a common cause of hyperuricemia. Nat Commun.

[6] Woodward OM, Kottgen A, Coresh J, Boerwinkle E, Guggino WB, Kottgen M (2009). Identification of a urate transporter, ABCG2, with a common functional polymorphism causing gout. Proc Natl Acad Sci USA.

[7] Hsu SP, Pai MF, Peng YS, Chiang CK, Ho TI, Hung KY (2004). Serum uric acid levels show a 'J-shaped' association with all-cause mortality in haemodialysis patients. Nephrol Dial Transplant.

[8] Latif W, Karaboyas A, Tong L (2011). Uric acid levels and all-cause and cardiovascular mortality in the hemodialysis population. Clin J Am Soc Nephrol.

[9] Culleton BF, Larson MG, Kannel WB, Levy D (1999). Serum uric acid and risk for cardiovascular disease and death: the Framingham Heart Study. Ann Intern Med.

[10] Niskanen LK, Laaksonen DE, Nyyssonen K (2004). Uric acid level as a risk factor for cardiovascular and all-cause mortality in middle-aged men: a prospective cohort study. Arch Intern Med.

[11] Matsuo H, Tsunoda T, Ooyama K (2016). Hyperuricemia in acute gastroenteritis is caused by decreased urate excretion via ABCG2. Sci Rep.

[12] Takada T, Yamamoto T, Matsuo H (2018). Identification of ABCG2 as an exporter of uremic toxin indoxyl sulfate in mice and as a crucial factor influencing CKD progression. Sci Rep.

[13] Shen S, Callaghan D, Juzwik C, Xiong H, Huang P, Zhang W (2010). ABCG2 reduces ROS-mediated toxicity and inflammation: a potential role in Alzheimer's disease. J Neurochem.

[14] Poller B, Drewe J, Krahenbuhl S, Huwyler J, Gutmann H (2010). Regulation of BCRP (ABCG2) and P-glycoprotein (ABCB1) by cytokines in a model of the human blood–brain barrier. Cell Mol Neurobiol.

[15] Ding R, Jin S, Pabon K, Scotto KW (2016). A role for ABCG2 beyond drug transport: regulation of autophagy. Autophagy.

[16] Nakashima A, Ohkido I, Yokoyama K, Mafune A, Urashima M, Yokoo T (2015). Proton pump inhibitor use and magnesium concentrations in hemodialysis patients: a cross-sectional study. PLoS ONE.

[17] Nakashima A, Ohkido I, Yokoyama K, Mafune A, Urashima M, Yokoo T (2017). Associations between low serum testosterone and all-cause mortality and infection-related hospitalization in male hemodialysis patients: a prospective cohort study. Kidney Int Rep..

[18] Matsuo H, Nakayama A, Sakiyama M (2014). ABCG2 dysfunction causes hyperuricemia due to both renal urate underexcretion and renal urate overload. Sci Rep.

[19] Yuan H, Yu C, Li X (2015). Serum uric acid levels and risk of metabolic syndrome: a dose–response meta-analysis of prospective studies. J Clin Endocrinol Metab.

[20] Antunovic T, Stefanovic A, Ratkovic M (2013). High uric acid and low superoxide dismutase as possible predictors of all-cause and cardiovascular mortality in hemodialysis patients. Int Urol Nephrol.

[21] Tsuruta Y, Nitta K, Akizawa T (2014). Association between allopurinol and mortality among Japanese hemodialysis patients: results from the DOPPS. Int Urol Nephrol.

[22] Beberashvili I, Erlich A, Azar A (2016). longitudinal study of serum uric acid, nutritional status, and mortality in maintenance hemodialysis patients. Clin J Am Soc Nephrol.

[23] Beberashvili I, Sinuani I, Azar A (2015). Serum uric acid as a clinically useful nutritional marker and predictor of outcome in maintenance hemodialysis patients. Nutrition.

[24] Park C, Obi Y, Streja E (2017). Serum uric acid, protein intake and mortality in hemodialysis patients. Nephrol Dial Transplant.

[25] Bae E, Cho HJ, Shin N (2016). Lower serum uric acid level predicts mortality in dialysis patients. Medicine.

[26] Glantzounis GK, Tsimoyiannis EC, Kappas AM, Galaris DA (2005). Uric acid and oxidative stress. Curr Pharm Des.

[27] Santos CX, Anjos EI, Augusto O (1999). Uric acid oxidation by peroxynitrite: multiple reactions, free radical formation, and amplification of lipid oxidation. Arch Biochem Biophys.

[28] Abuja PM (1999). Ascorbate prevents prooxidant effects of urate in oxidation of human low density lipoprotein. FEBS Lett.

[29] Ames BN, Cathcart R, Schwiers E, Hochstein P (1981). Uric acid provides an antioxidant defense in humans against oxidant- and radical-caused aging and cancer: a hypothesis. Proc Natl Acad Sci USA.

[30] Maliepaard M, Scheffer GL, Faneyte IF (2001). Subcellular localization and distribution of the breast cancer resistance protein transporter in normal human tissues. Cancer Res.

[31] Huls M, Brown CD, Windass AS (2008). The breast cancer resistance protein transporter ABCG2 is expressed in the human kidney proximal tubule apical membrane. Kidney Int.

[32] Nakayama A, Matsuo H, Nakaoka H (2014). Common dysfunctional variants of ABCG2 have stronger impact on hyperuricemia progression than typical environmental risk factors. Sci Rep.

[33] Sorensen LB (1965). Role of the intestinal tract in the elimination of uric acid. Arthr Rheum.

[34] Hosomi A, Nakanishi T, Fujita T, Tamai I (2012). Extra-renal elimination of uric acid via intestinal efflux transporter BCRP/ABCG2. PLoS ONE.

[35] Cao XS, Chen J, Zou JZ (2015). Association of indoxyl sulfate with heart failure among patients on hemodialysis. Clin J Am Soc Nephrol.

[36] Vanholder R, Schepers E, Pletinck A, Nagler EV, Glorieux G (2014). The uremic toxicity of indoxyl sulfate and p-crossly sulfate: a systematic review. J Am Soc Nephrol.

[37] Nangaku M, Mimura I, Yamaguchi J, Higashijima Y, Wada T, Tanaka T (2015). Role of uremic toxins in erythropoiesis-stimulating agent resistance in chronic kidney disease and dialysis patients. J Renal Nutr.

[38] Choi HK, Curhan G (2007). Independent impact of gout on mortality and risk for coronary heart disease. Circulation.

[39] Krishnan E, Svendsen K, Neaton JD, Grandits G, Kuller LH (2008). Long-term cardiovascular mortality among middle-aged men with gout. Arch Intern Med.

[40] McAdams-DeMarco MA, Maynard JW, Coresh J, Baer AN (2012). Anemia and the onset of gout in a population-based cohort of adults: Atherosclerosis Risk in Communities study. Arthr Res Therapy.

[41] Lanaspa MA, Sanchez-Lozada LG, Choi YJ (2012). Uric acid induces hepatic steatosis by generation of mitochondrial oxidative stress: potential role in fructose-dependent and -independent fatty liver. J Biol Chem.

[42] Sautin YY, Nakagawa T, Zharikov S, Johnson RJ (2007). Adverse effects of the classic antioxidant uric acid in adipocytes: NADPH oxidase-mediated oxidative/nitrosative stress. Am J Physiol Cell Physiol.

[43] Jia Y, Chen J, Zhu H, Jia ZH, Cui MH (2015). Aberrantly elevated redox sensing factor Nrf2 promotes cancer stem cell survival via enhanced transcriptional regulation of ABCG2 and Bcl-2/Bmi-1 genes. Oncol Rep.

[44] Silva-Palacios A, Konigsberg M, Zazueta C (2016). Nrf2 signaling and redox homeostasis in the aging heart: A potential target to prevent cardiovascular diseases?. Ageing Res Rev.

[45] Sperati CJ, Parekh RS, Berthier-Schaad Y (2009). Association of single-nucleotide polymorphisms in JAK3, STAT4, and STAT6 with new cardiovascular events in incident dialysis patients. Am J Kidney Dis.

[46] Friedman DJ, Afkarian M, Tamez H (2009). Klotho variants and chronic hemodialysis mortality. J Bone Miner Res.

[47] Rothuizen TC, Ocak G, Verschuren JJ (2015). Candidate gene analysis of mortality in dialysis patients. PLoS ONE.

