# Supplementary material for: Dysfunctional ABCG2 gene polymorphisms are associated with serum uric acid levels and all-cause mortality in hemodialysis patients
Source: Hum Cell. 2020 Mar 16;33(3):559–68. doi: 10.1007/s13577-020-00342-w (PMC7324430; doi:10.1007/s13577-020-00342-w)
Supplement: Supplementary file 1 — Supplementary file1 (DOCX 21 kb) [file 13577_2020_342_MOESM1_ESM.docx]

Supp 1. Patients characteristics divided by ABCG2 function types

|  | ABCG2 function types | | | | | | | | |
| --- | --- | --- | --- | --- | --- | --- | --- | --- | --- |
|  | Full function | | 3/4 function | | 1/2 function | | ≤ 1/4 function | | *P*-value |
| Number | 568 | | 504 | | 129 | | 13 | |  |
| Age (yr) | 63 | (12.0) | 64 | (11.6) | 64 | (11) | 67 | (8.9) | 0.219 |
| Male (%) | 395 | (69.5) | 355 | (70.4) | 91 | (70.5) | 7 | (53.9) | 0.658 |
| Dialysis vintage (mo) | 90 | (40-155) | 83 | (39-147) | 80 | (37-146) | 125 | (85-234) | 0.145 |
| Diabetes mellitus (%) | 223 | (39.3) | 185 | (36.7) | 50 | (38.8) | 2 | (15.4) | 0.262 |
| Body mass index (kg/m^2^) | 22.0 | (5.0) | 21.8 | (5.2) | 21.0 | (4.5) | 20.1 | (3.2) | 0.186 |
| sBP (mmHg) | 153 | (22) | 151 | (22) | 151 | (21) | 151 | (23) | 0.338 |
| dBP (mmHg) | 80 | (15) | 78 | (13) | 79 | (14) | 80 | (14) | 0.21 |
| Hemoglobin (g/dl) | 10.6 | (1.1) | 10.4 | (1.0) | 10.4 | (1.2) | 10.5 | (1.2) | 0.118 |
| Albumin (g/dl) | 3.8 | (0.4) | 3.8 | (0.4) | 3.8 | (0.39) | 3.7 | (0.4) | 0.974 |
| Blood urea nitrogen (mg/dl) | 65 | (14) | 65 | (14) | 66 | (15) | 55 | (12) | 0.061 |
| Creatinine (mg/dl) | 11.7 | (3.2) | 11.5 | (3.0) | 11.5 | (2.6) | 11.6 | (5.2) | 0.893 |
| Uric acid (mg/dl) | 7.4 | (1.2) | 7.9 | (1.3) | 8.2 | (1.4) | 8.7 | (1.3) | <0.001 |
| < 6.8 mg/dl | 173 | (30.5) | 107 | (21,2) | 16 | (12.4) | 1 | (7.7) | <0.001 |
| 6.8-7.6 mg/dl | 169 | (29.7) | 128 | (25.4) | 33 | (25.6) | 2 | (15.4) | <0.001 |
| 7.6-8.5 mg/dl | 126 | (22.2) | 116 | (23.0) | 27 | (20.9) | 4 | (30.8) | <0.001 |
| ≥ 8.5 mg/dl | 100 | (17.6) | 153 | (30.4) | 53 | (41.1) | 6 | (46.1) | <0.001 |
| Sodium (mEq/L) | 139 | (3) | 139 | (3) | 139 | (3) | 138 | (4) | 0.026 |
| Potassium (mEq/L) | 4.98 | (0.7) | 5.02 | (0.72) | 5.03 | (0.739 | 4.74 | 0.75 | 0.478 |
| ALP (IU/L) | 218 | (173-278) | 218 | (171-283) | 207 | (174-253)) | 217 | (160-293) | 0.321 |
| Calcium (mg/dl) | 8.9 | (0.7) | 8.9 | (0.6) | 8.9 | (0.6) | 9.0 | (0.6) | 0.72 |
| Phosphate (mg/dl) | 5.5 | (1.4) | 5.4 | (1.3) | 5.4 | (1.5) | 5.5 | (1.0) | 0.617 |
| Magnesium (mg/dl) | 2.6 | (0.4) | 2.6 | (0.5) | 2.5 | (0.5) | 2.4 | (0.3) | 0.277 |
| iPTH (pg/ml) | 143 | (83-240) | 148 | (86-218) | 147 | (83-233) | 128 | (65-225) | 0.454 |
| C-reactive protein (mg/dl) | 0.1 | (0.06-0.38) | 0.1 | (0.05-0.34) | 0.1 | (0.05-0.35) | 0.2 | (0.06-0.53) | 0.782 |
| Kt/V | 1.4 | (0.5) | 1.4 | (0.3) | 1.4 | (0.3) | 1.5 | (0.3) | 0.542 |
| Anti-hyperuricemic drug (%) | 71 | (12.5) | 90 | (17.9) | 35 | (27.1) | 2 | (15.4) | <0.001 |
| Antiplatelet drug (%) | 274 | (48.2) | 248 | (49.2) | 53 | (41.1) | 4 | (30.8) | 0.227 |
| Anticoagulant drug (%) | 34 | (6.0) | 47 | (9.3) | 11 | (8.5) | 0 | (0) | 0.091 |
| ACE-I or ARB (%) | 260 | (46.8) | 254 | (51.9) | 76 | (60.3) | 8 | (61.5) | 0.029 |
| Statin (%) | 149 | (26.2) | 134 | (26.6) | 28 | (21.7) | 2 | (15.4) | 0.53 |
| Past history |  |  |  |  |  |  |  |  |  |
| Cardiovascular disease (%) | 98 | (17.3) | 108 | (21.4) | 14 | (10.9) | 2 | (15.4) | 0.028 |
| Malignancy (%) | 97 | (17.1) | 78 | (15.5) | 24 | (18.6) | 3 | (23.1) | 0.733 |

Data are means (SD), N(%), or medians (interquartile range) as appropriate.

Abbreviations: UA, uric acid; sBP, systolic blood pressure; dBP, diastolic blood pressure; ALP, alkaline phosphatase; iPTH, intact parathyroid hormone; ACE-I, angiotensin converting enzyme inhibitor; ARB, angiotensin II receptor blocker
